# Trocar number and placement for laparoscopic sleeve gastrectomy and comparison of single-incision and conventional laparoscopic sleeve gastrectomy: a systematic review and meta-analysis

**DOI:** 10.1097/JS9.0000000000000402

**Published:** 2023-04-18

**Authors:** Zhengchen Jiang, Zhao Zhang, Tianyi Feng, Yugang Cheng, Guangyong Zhang, Mingwei Zhong, Sanyuan Hu

**Affiliations:** a Department of General Surgery, Shandong Provincial Qianfoshan Hospital, Cheeloo College of Medicine, Shandong University; b Department of General Surgery, The First Affiliated Hospital of Shandong First Medical University, Shandong Provincial Qianfoshan Hospital, Jinan, Shandong Province, China

**Keywords:** conventional laparoscopic sleeve gastrectomy, meta-analysis, single-incision laparoscopic sleeve gastrectomy, systematic review, trocar placement

## Abstract

Conventional laparoscopic sleeve gastrectomy (CLSG) has been conducted in multiple centers for treating morbid obesity, however, there are no standard criteria for (1) placing the trocar; and (2) how many trocars should be used. Single-incision laparoscopic sleeve gastrectomy (SLSG), a newly emerged technique in 2008, has been proposed as an alternative to CLSG in recent years, however, there is no definite evidence for this.

**Materials and methods:**

A systematic literature search was performed using the PubMed, Embase, Web of Science, and Cochrane Library databases for laparoscopic sleeve gastrectomy cases from January 2006 to October 2022. We then summarized the trocar numbers and placement patterns among these studies. A meta-analysis was conducted to compare the difference between SLSG and CLSG in the perioperative and postoperative indices.

**Results:**

A total of 61 studies involving 20 180 patients who underwent laparoscopic sleeve gastrectomy for treating morbid obesity were included in the systematic review, including 11 on SLSG, 35 on CLSG, and 15 studies comparing SLSG and CLSG. A systematic review showed that the trocar number varied in different CLSG studies, mainly using four or five trocars. The trocars were mainly placed in position, presenting an inverted trapezoid pattern and a left-predominant pattern. Meta-analysis showed that the operative time in the SLSG was significantly higher than that in the CLSG, and the pain Visual Analog Scale rating on postoperative day 1 in the CLSG was significantly higher than in the SLSG. There were no statistical significances in the other complications or surgical efficiency.

**Conclusions:**

In the CLSG, the majority of the trocars were arranged in an inverted trapezoid pattern and were of the left-predominant type. Although SLSG is a feasible technique in selected patients, there is insufficient evidence to recommend its widespread use compared with CLSG. High-quality randomized controlled trials with large study populations and long follow-up periods will be required in the future.

## Introduction

Highlights

- In conventional laparoscopic sleeve gastrectomy (CLSG) studies, the majority was performed using four and five trocars, especially the latter.
- In the single-incision laparoscopic sleeve gastrectomy (SLSG) studies, the majority utilized periumbilical trocar placement.
- In the CLSG, most of the trocars were arranged in an inverted trapezoid (IT) pattern and left-predominant type.
- Compared with CLSG, SLSG indicated higher operative time and lower postoperative pain.

Laparoscopic sleeve gastrectomy (LSG) is a restrictive procedure designed to decrease appetite by reducing gastric distension and producing a sensation of fullness. It has been commonly adopted in bariatric surgery for treating morbid obesity^1^. It can be performed laparoscopically, even in patients with massive obesity, requiring only a short hospital stay. Indeed, surgical procedures in morbidly obese patients are surgically more challenging, especially in the central obesity (apple-shaped) patients with a higher BMI in the male population, yielding a prolonged operative time^2^.

Appropriate trocar placement contributes to the laparoscopic vision of target organs and tissues, which facilitates the optimal vision of the operative field and decreases mental and muscular fatigue for the surgeons. In addition, it can enhance the recognition of anatomical structures and pathological conditions^3^. Most surgeons have probably been in a dilemma as there is a negative visualization due to inappropriate trocar placement, such as trocar clashing. In a questionnaire-based survey involving 370 bariatric and metabolic surgeons from 59 countries, inappropriate trocar placement was considered the most challenging factor for LSG^4^. CLSG requires three to five skin incisions to place the trocar^5^, and occasionally additional trocars are needed intraoperatively based on the surgical fields and operation. Attempts (e.g. occupational biomechanics) have been made to determine the optimal method for trocar placement in laparoscopic bariatric surgeries. For example, the distance between the xiphoid process and the umbilicus, designated as the XU distance, was considered a key element in determining the choice of trocar placement for laparoscopic bariatric surgery^2^.

Since the emergence of SLSG in 2008, an increasing number of morbidly obese patients have chosen to undergo such surgery because it has certain advantages such as improved cosmesis and reduced postoperative pain^6^. As a newly emerging surgery, more attention has been paid to the safety of these procedures. Although SLSG can be conducted with conventional 10-mm front-view laparoscopes and straight instruments, the freedom of motion of the surgeon and assistant was significantly reduced in the presence of crowding over the working area. Moreover, the limited triangulation increases the difficulty of tissue exposure and dissection^7^. Recently, although the triangulation problem was compensated by the newly developed single-incisional laparoscopy, it involves a high learning curve, a longer operative time, and potential anesthetic injuries^8^.

Most studies on trocar placement are based on the personal experiences of the surgeons or a single center, with a lack of consensus on the placement standards. In addition, there are still disputes regarding whether SLSG could replace the CLSG for treating morbid obesity in terms of safety and efficiency. To date, only two meta-analyses have compared partial of the operative indices (e.g. operative time) between SLSG and CLSG, rather than the incisional hernia, gastroesophageal reflux disease (GERD), and excess weight loss (EWL) at 12 and 24 months. Unfortunately, there is a lack of high-quality cohort studies comparing CLSG and SLSG. This study was designed to illustrate the trocar placement and number based on a systematic analysis, and simultaneously, a meta-analysis was carried out to compare the safety and feasibility of SLSG and CLSG.

## Materials and methods

### Study design

According to the Preferred Reporting Items for Systematic Reviews and Meta-Analyses (PRISMA)^9^ and meta-analyses of observational studies in epidemiology, we prepared a protocol consisting of a search strategy, inclusion and exclusion criteria, primary and secondary outcomes, and statistical analysis. This study was registered in the PROSPERO. This work has been reported in line with PRISMA and Assessing the Methodological Quality of Systematic Reviews (AMSTAR) Guidelines.

### Literature search strategy

We searched PubMed, Embase, Web of Science, and Cochrane Library databases. We used the following terms in every possible combination: MeSH ‘bariatric surgery,’ the word roots ‘laparoscope,’ and ‘laparoendoscope,’ and the keywords ‘sleeve gastrectomy,’ ‘site location,’ ‘port location,’ ‘trocar location,’ ‘site number,’ ‘port number.’ Manual research was conducted to extend this search. The inclusion criteria were as follows: (1) articles published between January 2006 and October 2022; (2) articles published in English; (3) studies reporting the subject information; (4) studies reporting the comparison between CLSG and SLSG; and (5) studies reporting the trocar placement information. The exclusion criteria were as follows: (1) meeting abstracts, reviews, case reports, and clinical guidelines; (2) other bariatric surgeries or revision, or conversion procedures; (3) studies with inadequate information; and (4) duplicate studies. Two authors performed a literature search. Disagreements were settled through detailed discussions with an experienced member of staff.

### Comparison of surgical indices

The surgical indices between the CLSG and SLSG included operative time, estimated blood loss, length of stay, postoperative analgesia [including pain Visual Analog Scale (VAS) ratings on postoperative day (POD) 1, 2, and 3], leak, postoperative bleeding, reoperation, stricture/obstruction, wound problem, incisional hernia, GERD, and EWL.

### Data extraction

Two authors extracted the data from the studies. The extracted data included sample size, sex, age, type of surgery, trocar utilization, trocar placement, operative time, estimated blood loss, length of stay, pain VAS score, leak, postoperative bleeding, reoperation, stricture/obstruction, wound problem, incisional hernia, GERD, and EWL. In cases of any disputes between them, detailed communication was held together with an experienced staff until consensus.

### Risk of bias assessment

The methodological quality of the included non–randomized controlled trial (RCT) studies was determined by the Newcastle–Ottawa scale (NOS). A study was assessed as low quality if the score was less than 5. A study with a NOS of 5 or more was assessed as high quality and was included in our meta-analysis.

### Data analysis

Continuous and dichotomous variables were analyzed based on weighted mean difference and odds ratio. If the values of the obtained continuous variables were represented as mean-cross-checked and median-cross-checked with maximum and minimum values, the mean and SD were obtained based on the previous description^10,11^. Heterogeneity among the studies was evaluated using the χ^2^ and *I* ^2^ statistics. For studies with an obvious heterogeneity and a *P*-value of more than 0.1, the random-effects model was adopted. Egger’s test and funnel plot analyses were used to evaluate publication bias. The analyses were performed using Review Manager, version 5.4.

## Results

### Overview of the studies involving the trocar number based on a systematic review

**Figure 1:**
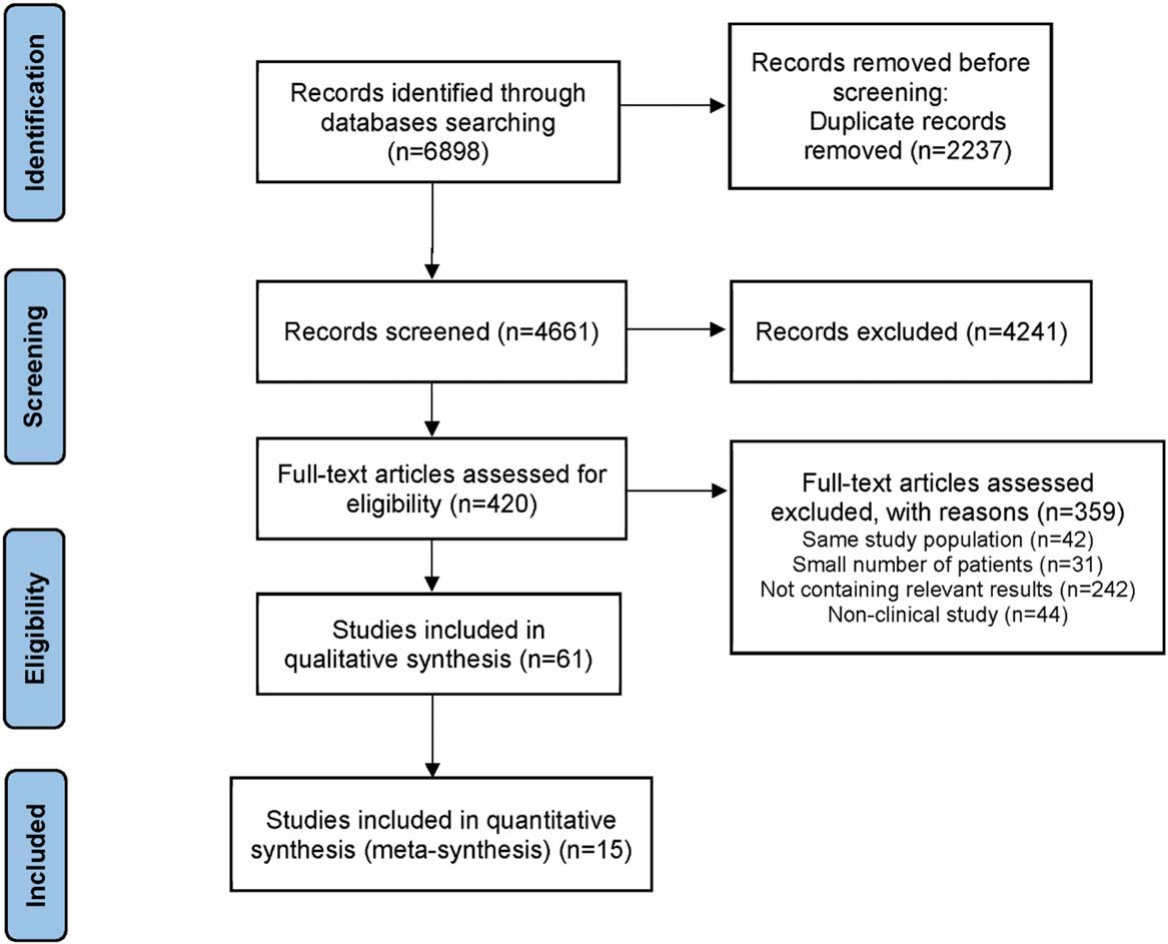
Flow diagram of the systematic literature search.

A flowchart diagram of the literature search is shown in Figure 1. A total of 6898 articles were screened from the PubMed, Embase, Web of Science, and Cochrane Library databases, and 61 studies reporting trocar numbers were included in the systematic review, including 11 studies^6,12–21^ on SLSG (Table 1), 35 studies^5,22–56^ on CLSG (Table 2), and 15 studies^57–71^ comparing SLSG and CLSG (Table 3). Among the 11 studies in Table 1, 10 studies utilized one trocar for SLSG, and one study used two trocars for the procedures. As shown in Table 2, the majority of CLSG was conducted using four (eight studies) or five trocars (19 studies), while the others were performed with three trocars in four studies (including one study using three to four trocars), six trocars in two studies, and seven trocars in two studies. As shown in Table 3, most of the studies were performed based on the comparison of SLSG and CLSG.

**Table 1:** Baseline characteristics of the included studies involved single-incision laparoscopic sleeve gastrectomy

References	Trocar number	Cases (female)	Age (years)	Preoperative BMI (kg/m^2^)	Operative time (min)	Estimated blood loss (ml)	Length of stay (days)	EWL at postoperative 6 months (%)	EWL at postoperative 12 months (%)	EWL at postoperative 24 months (%)
Saber *et al.* ^6^	1	7 (4)	45.6 (29–62)	53.5 (42–68)	125 (90–177)	–	2.4 (1–3)	–	–	–
Gentileschi *et al.* ^12^	1	8 (5)	44.4 (39–52)	56.2 (44.2–62.6)	128 (84–140)	–	2.4 (1–3)	–	–	–
Taller *et al.* ^13^	1	10 (10)	37.6 (26–52)	41.2 (35–51)	93 (61–144)	25 (10–50)	1.8 (1–3)	–	–	–
Galvani *et al.* ^14^	1	8 (8)	34.1 (24–48)	49 (39–64)	103.8 (75–150)	–	2	–	–	–
Alevizos and Lirici^15^	1	12 (9)	44.5 (28–60)	41.8 (37–50)	151.7 (100–185)	–	6.5 (5–12)	–	71.3 (22–86)	–
Ghinagow *et al.* ^21^	1	51 (38)	31±7.7	36.32±2.89	72±27.91	–	2±0.84	–	76.12±17.54	–
Maluenda *et al.* ^16^	1	20 (20)	34.5 (21–57)	35.1 (30.5–40)	127 (90–170)	–	–	–	–	–
Fernández *et al.* ^17^	1	74 (72)	34.2±9.2	34.0±3.2	48±10	–	2.4±2.0	–	–	–
Gaillard *et al.* ^18^	1	1000 (845)	40.1±12.3	42.6 (33.8–84.6)	112 (50–360)	–	4 (2–125)	–	69.0±24.5	62.2±27.4
Lainas *et al.* ^19^	1	1800 (NA)	42.3	45.3	88	–	4 (1–125)	–	71.1	73.7
Farías *et al.* ^20^	2	237 (221)	36±10.2	33.5±3.3	49.5±14.9	–	2.2±1	107±41	116±38	–

Values are represented as Means±SD or Means (Minimum–Maximum).

EWL, excess weight loss, NA, not available.

**Table 2:** Baseline characteristics of the included studies involved conventional laparoscopic sleeve gastrectomy

References	Trocar number	Cases (female)	Age (years)	Preoperative BMI (kg/m^2^)	Operative time (min)	Length of stay (days)	EWL at postoperative 6 months (%)	EWL at postoperative 12 months (%)	EWL at postoperative 24 months (%)
Noel *et al.* ^32^	3	750 (570)	41.2	43.7	–	–	59	73	73
Jarallah *et al.* ^45^	3	808 (642)	28.34±8.37	41.09±6.34	43±20 (31–185)	2 (1–5)	55.08±22.9	82.6±22.6	–
Li *et al.* ^49^	3	207 (85)	34.50±9.76	40.08±6.65	167.26±49.04	5.07±5.36	57.97±19.28	66.08±25.42	–
Aridi *et al.* ^47^	3–4	400 (240)	36.4±12.7	42.3±6.6	107.9±20.6	2.4±1.2	–	–	83
Fuks *et al.* ^23^	4	135 (113)	40 (18–65)	48.8 (37–72)	103 (30–550)	3.8	38.6	49.4	56
Chopra *et al.* ^28^	4	174 (149)	39.59±10.71	48.97±8.25	103.9 (40–270)	2.6 (2–25)	44.76±11.91	55.52±17.59	59.22±18.26
Gibson *et al.* ^34^	4	500 (340)	41 (17–73)	45 (35–76)	–	3.8 (3–12)	58	76	71
Obeidat and Shanti^39^	4	190 (NA)	34.0±10.8	46.2±7.7	90.4±44.1	2.5±2.2	60.1±20.1	75.1±22.8	72.6±17.5
Sakran *et al.* ^40^	4	3003 (1901)	43 (14–73)	42.8	50 (32–94)	2.2 (1–38)	–	72	–
Al-Mulhim^42^	4	112 (73)	26 (20–37)	41 (35–52)	151 (95–190)	4 (3–8)	–	43 (33–51)	–
Sepúlveda *et al.* ^44^	4	1023 (688)	40.6±10.8	37±4.5	67.6±23.4	–	–	–	–
Garg *et al.* ^48^	4	424 (284)	39.8±11.2	46.67±7.9	–	–	–	71.8±25.3	70.6±20.5
Gadiot *et al.* ^29^	5	445 (335)	42 (18–63)	46±6	41 (19–196)	2 (1–61)	–	71±26	69±28
Chowbey *et al.* ^24^	5	75 (40)	44.4 (19–67)	58.0 (33.28–77.3)	60 (40–90)	4 (3–6)	52.3 (44.3–58)	59.13 (46–69.3)	65.2 (51.1–70.2)
Bellanger and Greenway^25^	5	529 (431)	43.43±10.63	44.26±8.54	–	–	42.36	65.92	66.11
D’Hondt *et al.* ^26^	5	83 (61)	40.4 (18–76)	39.3 (35–52)	–	4.93 (4–8)	–	78.5 (26–129)	72 (46–113)
Angrisani *et al.* ^27^	5	121 (78)	38.8±10.9	48.7±9.3	105 (95–180)	5.6 (1–14)	48.1±19.3	51.7±25.0	53.1±16.6
Prasad *et al.* ^30^	5	110 (84)	39.3±11.15	44.58±6.82	64.81±10.62	3.95±0.73	53.15±11.79	67.57±13.01	71.19±13.86
Boza *et al.* ^31^	5	1000 (773)	36.9±11.5	37.4±4.0	70 (30–360)	3 (2–16)	81	86.6	84.1
Zachariah *et al.* ^23^	5	228 (145)	34.68±10.1	37.42±4.75	60.63±27.37	1.08±1.01	–	72.39±16.00	72.63±18.07
Schraibman *et al.* ^35^	5	32 (16)	46±13	39.4±3.8	138 (115–170)	3 (2–4)	–	–	–
Chaar *et al.* ^36^	5	338 (266)	45.30±11.46	44.49±6.28	–	29.18±11.64	57.51±14.95	64.10±17.98	56.91±21.31
Seki *et al.* ^38^	5	179 (89)	40.7±11.2	43.3±10.0	140±37	3.3±1.1	–	68.5±24.3	72.9±23.8
Yildiz *et al.* ^41^	5	159 (131)	39.6±9.4	47.5±5.8	105±41	7.3±4.2	53.1±12.3	70.1±11.7	75.1±10.5
Altun *et al.* ^43^	5	750 (171)	37.4±9.8	42.8±7.7	51±17.9	3	76.9±20.9	85.5±23.6	89.7±27.6
Hans *et al.* ^46^	5	218 (150)	29.9±7.3	38.3±6.2	103.0±23.1	3.7±0.9	58.2±11.1	62.8±16.9	49.5±18.5
Lemaître *et al.* ^54^	5	494 (367)	45.5 (18–75)	46.2±6.4	60 (40–90)	5 (3–21)	–	31.5±5.8	31.3±6.4
Biter *et al.* ^55^	5	76 (63)	45.5±11.2	44.17±5	–	–	–	73.21 ±22.82	–
Misra *et al.* ^50^	5	284 (159)	41.08±12.02	44.9±7.9	52±17.8	2.6±1.2	–	76.3±25.5	–
Nasta *et al.* ^52^	5	218 (133)	39.2±11.8	45.4±9.4	–	–	–	87.6±28.9	77.3±29.3
Toolabi *et al.* ^53^	5	750 (601)	37.9±10.2	40.2±6.0	–	–	–	77.6±1.0	75.8
Bhandari *et al.* ^51^	6	152 (81)	40.4±13.2	45.0±8.4	46±7	6.7±1.5	–	–	–
Park and Kim^37^	6	192 (131)	33.1±9.6	40.0±7.2	104.4±28.1	2.2±5.6	60.8±22.6	72.6±25.1	80.6±19.8
Moy *et al.* ^22^	7	135 (75)	43.5 ±12.8	60.1±10.6	134	2	39.9±11.3	47.3±16	–
Roa *et al.* ^5^	7	30 (23)	40 (17–69)	41.4 (33–59)	80 (65–130)	3.2 (2–25)	52.8	–	–

Values are represented as Means±SD or Means (Minimum–Maximum).

EWL, excess weight loss.

**Table 3:** Basic characteristics of studies involved the comparison of SLSG versus CLSG

			Case number (female)		Age		Preoperative BMI (kg/m^2^)		Operative time (min)		Estimated blood loss (ml)		Length of stay (days)		EWL at postoperative 6 months (%)		EWL at postoperative 12 months (%)		EWL at postoperative 24 months (%)	
References	Type of comparison	NOS score	SLSG	CLSG	SLSG	CLSG	SLSG	CLSG	SLSG	CLSG	SLSG	CLSG	SLSG	CLSG	SLSG	CLSG	SLSG	CLSG	SLSG	CLSG
Saber *et al.* ^57^	1/NA	7	14 (7)	12 (7)	44.2±11	43.1±13.6	53.8±6.6	52.6±6.8	128±19.3	110±33.4	7.8±36.6	93±29.9	1.7±0.7	2.3±0.7	–	–	–	–	–	–
Park *et al.* ^58^	1/NA	7	25 (23)	9 (8)	46.8±13.3	47.9±14	47.1±6.6	48.5±9.3	118.4±30.7	101.1±31.3	–	–	59.1±17.1	62.7±12.8	–	–	–	–	–	–
Nguyen *et al.* ^59^	1/5	7	26 (17)	24 (16)	44±11	47±11	42±4	47±7	78±26	84±24	23±14	23±14	1.4±0.6	1.8±0.7	–	–	–	–	–	–
Delgado *et al.* ^60^	1/5	6	20 (15)	22 (15)	46 (20–66)	50 (26–69)	40.1 (35.6–55.6)	40.6 (34.5–55.3)	79.2 (50–130)	54.1 (40–90)	–	–	2.75	–	60.12/61.86	–	–	–	–	–
Sucher *et al.* ^61^	1/4	8	40 (0)	40 (0)	37 (19–62)	43 (24–73)	40.8 (35.1–45.0)	43.8 (35.0–47.8)	84.8±21.3	97.4±26.0	–	–	5 (4–24)	6 (4–14)	–	–	–	–	–	–
Gomberawalla *et al.* ^62^	1/4	8	36 (34)	36 (29)	43.33 (27–62)	46 (31–72)	43.06 (37–48)	43.72 (34–50)	116.78 (79–197)	118.25 (57–218)	–	–	1.80 (1–3)	1.75 (1–3)	58.4 (38–102)	58.5 (44–95)	72.3 (24–125)	74.1 (33–108)	–	–
Lakdawala *et al.* ^63^	1/5	8	300 (279)	300 (150)	35.5±9.7	35.5±7.8	39.9±5.2	39.9±5.1	45±20.5	42±18.2	–	–	–	–	59.7±19.7	58.6±22.3	69.2±22.8	68.3±24	65.4±29.6	69.1±26.3
Muir and Rice^64^	1/5	7	32 (31)	30 (27)	32.4	35	42.1	46.5	104.6 (68–165)	90.7 (40–139)	32.1 (10–70)	34.3 (10–75)	–	–	48.3	59.1	–	–	–	–
Porta *et al.* ^65^	1/5	6	65 (53)	65 (51)	36±2.9	39±2.3	40.09±0.3	41.01±0.4	60±15	61±11	–	–	–	–	48.9±3.1	48.3±2.9	56.9±2.9	56.5±3.2	59.6±4.6	59.9±4.1
Morales-Conde *et al.* ^66^	1/5	8	15 (13)	15 (15)	41.2 (27–58)	46.8 (23–62)	44.35 (39.9–49.1)	45.52 (41.0–49.5)	69.93 (51–88)	61.80 (36–92)	7.66 (0–40)	17.80 (0–150)	–	–	56.9±14.9	53.3±13.7	–	–	–	–
Mauriello *et al.* ^67^	1/3	8	90 (80)	187 (106)	45±12	52±15	45.3±2.4	53.3±3.6	86±16	72±15	45±16	22±12	3±2	5±3	59.2±19.3	59.7±19.9	66.1±24.6	65.1±24.4	67.5±27.2	67.2±26.8
Amiki *et al.* ^68^	2/5	7	31 (NA)	31 (NA)	39.1±8.7	39.7±9.0	33.5±2.6	33.7±2.0	148.7±22.6	120.2±25.9	–	–	–	–	–	–	105.9±39.3	109.7±40.0	101.1±51.9	105.3±60.6
Tranchart *et al.* ^69^	1/4	7	314 (253)	314 (246)	41 (32–49)	41 (32–50)	42.8 (39.7–47.0)	42.7 (39.8–46.7)	65 (55–80)	50 (45–60)	–	–	3 (3–3)	2 (2–2)	69.5 (53.2–93.6)	73.3 (60–92.3)	69.8 (54.8–87.3)	73.4 (58.1.9–95.7)	–	–
Khidir *et al.* ^70^	1/NA	8	200 (153)	220 (121)	33.3±10.5	34.7±16.5	43.8±5.6	48.6±8.1	74.5±26	68.3±26	–	–	3.4±0.9	4±1	–	–	67.2±18.7	67.7±23.1	–	–
Park *et al.* ^71^	1–2/4	8	75 (61)	73 (35)	38.0±11.4	38.3±11.5	37.7±5.1	40.9±5.8	–	–	–	–	–	–	80.3±25.3	65.9±26.5	90.0±29.8	75.2±29.9	–	–

Values are represented as Means±SD or Means (Minimum–Maximum).

CLSG, conventional laparoscopic sleeve gastrectomy; EWL excess weight loss; NA not available; SLSG single-incision laparoscopic sleeve gastrectomy.

### Studies involving trocar placement in the systematic review

**Figure 2:**
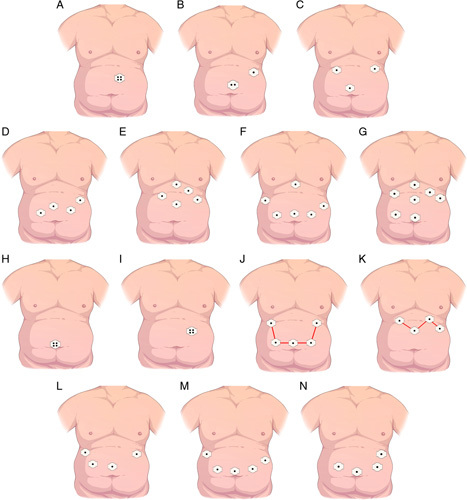
Schematic diagram illustrating the trocar placement. (A–G) Representative trocar placement from single-port to seven-port. Schematic diagram of periumbilical (H) and left upper abdominal (I) trocar placement of single-incision laparoscopic sleeve gastrectomy. Schematic diagram of ‘inverted trapezoid’ shape (J) and ‘Z’ shape (K) trocar placement. Schematic diagram of right-predominant (L), central type (M), and left-predominant (N) trocar placement.

A diagram depicts the trocar placement in these 61 studies, illustrating the placement pattern of these trocars, involving one to seven trocars (Figs. 2A–G). As shown in Table 4, 26 studies illustrated trocar placement in SLSG, showing periumbilical and left upper abdominal locations (Figs. 2H and I), with the former being the predominant type. As shown in Table 5, 27 studies illustrated the exact trocar placement in CLSG. We summarized the preferred location sites for the trocars used by the surgeons, based on the shape of the trocar placement. The shape of the IT was designated for trocar placement in 19 studies as shown in Figure 2J, while a ‘Z’ shape was shown in four studies as shown in Figure 2K. The other four studies reported no regular shape for the trocar placement. Interestingly, not all trocars were equally distributed along these lines. According to the predominant body side of trocar placement, the trocar placement pattern could be divided into three types: right-predominant (one study), central (10 studies), and left-predominant (16 studies) (Figs. 2L and N), with the majority of trocars distributed on the left side.

**Table 4:** Studies that were included in the systematic review about trocar placement of single-incision laparoscopic sleeve gastrectomy

References	Country	Trocar (*n*)	Location of trocar
Saber *et al.* ^6^	USA	1	Umbilicus
Gentileschi *et al.* ^12^	Italy	1	Umbilicus
Saber *et al.* ^57^	USA	1	Umbilicus
Taller *et al.* ^13^	USA	1	Left upper quadrant
Galvani *et al.* ^14^	USA	1	Left upper quadrant
Park *et al.* ^58^	UK	1	Umbilicus
Alevizos and Lirici^15^	Italy	1	Umbilicus
Nguyen *et al.* ^59^	USA	1	Umbilicus
Delgado *et al.* ^60^	Spain	1	Umbilicus
Farías *et al.* ^20^	Chile	1	Umbilicus
Ghinagow *et al.* ^21^	China	1	Umbilicus
Sucher *et al.* ^61^	Austria	1	Umbilicus
Maluenda *et al.* ^16^	Chile	1	Umbilicus
Gomberawalla *et al.* ^62^	USA	1	Umbilicus
Fernández *et al.* ^17^	Chile	1	Umbilicus
Lakdawala *et al.* ^63^	India	1	Umbilicus
Muir and Rice^64^	USA	1	Umbilicus
Gaillard *et al.* ^18^	France	1	Left upper quadrant
Porta *et al.* ^65^	Italy	1	Umbilicus
Morales-Conde *et al.* ^66^	Spain	1	Umbilicus
Mauriello *et al.* ^67^	France	1	Umbilicus
Amiki *et al.* ^68^	Japan	1	Umbilicus
Tranchart *et al.* ^69^	France	1	Left upper quadrant
Khidir *et al.* ^70^	Qatar	1	Umbilicus
Park *et al.* ^71^	Korea	1	Umbilicus
Lainas *et al.* ^19^	France	1	Both

**Table 5:** Studies that were included in the systematic review about trocar placement of conventional laparoscopic sleeve gastrectomy

References	Country	Trocar (*n*)	Shape description of trocar placement	Predominant body side of trocar placement
Roa *et al.* ^5^	USA	7	Other	Middle
Moy *et al.* ^22^	USA	7	Other	Middle
Fuks *et al.* ^23^	France	4	IT	Left
Chowbey *et al.* ^24^	India	5	Z	Left
Bellanger and Greenway^25^	USA	5	IT	Middle
D’Hondt *et al.* ^26^	Belgium	5	IT	Middle
Angrisani *et al.* ^27^	Italy	5	IT	Left
Chopra *et al.* ^28^	USA	4	IT	Middle
Gadiot *et al.* ^29^	Netherlands	5	Z	Left
Prasad *et al.* ^30^	India	5	Z	Left
Noel *et al.* ^32^	France	3	IT	Left
Zachariah *et al.* ^23^	China	5	IT	Left
Schraibman *et al.* ^35^	Brazil	5	IT	Middle
Lakdawala *et al.* ^63^	India	1/5	IT	Left
Obeidat and Shanti^39^	Jordan	4	IT	Left
Yildiz *et al.* ^41^	Türkiye	5	Other	Left
Al-Mulhim^42^	Saudi Arabia	3	IT	Middle
Porta *et al.* ^65^	Italy	1/5	IT	Right
Morales-Conde *et al.* ^66^	Spain	1/5	IT	Left
Sepúlveda *et al.* ^44^	Chile	4	IT	Left
Biter *et al.* ^55^	The Netherlands	5	Z	Left
Mauriello *et al.* ^67^	France	1/3	IT	Middle
Li *et al.* ^49^	China	3	Other	Left
Amiki *et al.* ^68^	Japan	2/5	IT	Left
Bhandari *et al.* ^51^	India	6	IT	Middle
Lemaître *et al.* ^54^	France	5	IT	Left
Tranchart *et al.* ^69^	France	1/4	IT	Middle

IT, inverted trapezoid; other; no regular trocar placement.

### Meta-analysis for the single-incision laparoscopic sleeve gastrectomy and conventional laparoscopic sleeve gastrectomy

Finally, 15 studies^57–71^ were included in this meta-analysis, and all showed a NOS of more than 5 (Table 3). We then analyzed the operative time, estimated blood loss, leak, postoperative bleeding, reoperation, stricture/obstruction, wound problem, incisional hernia, GERD, length of hospital stay, pain VAS rating on POD 1, 2, and 3, and EWL at 6, 12, and 24 months postoperatively (Tables 6 and 7).

**Table 6:** Summary of the operative technical details of studies that were included in meta-analysis

	Operative time (min)		Estimated blood loss (ml)		Length of stay (days)		VAS at POD 1		VAS at POD 2		VAS at POD 3		EWL at postoperative 6 months		EWL at postoperative 12 months		EWL at postoperative 24 months	
References	SLSG	CLSG	SLSG	CLSG	SLSG	CLSG	SLSG	CLSG	SLSG	CLSG	SLSG	CLSG	SLSG	CLSG	SLSG	CLSG	SLSG	CLSG
Saber *et al.* ^57^	128±19.3	110±33.4	7.8±36.6	93±29.9	1.7±0.7	2.3±0.7	NA	NA	NA	NA	NA	NA	NA	NA	NA	NA	NA	NA
Park *et al.* ^58^	118.4±30.7	101.1±31.3	NA	NA	2.46±0.71	2.61±0.53	0.6±1.0	0.3±0.7	NA	NA	NA	NA	NA	NA	NA	NA	NA	NA
Nguyen *et al.* ^59^	84±24	78±26	30±21	23±14	1.8±0.7	1.4±0.6	NA	NA	NA	NA	NA	NA	NA	NA	NA	NA	NA	NA
Delgado *et al.* ^60^	79.2 (50–130)	54.1 (40–90)	NA	NA	2.75	NA	NA	NA	NA	NA	NA	NA	60.12	61.86	NA	NA	NA	NA
Sucher *et al.* ^61^	84.8±21.3	97.4±26.0	NA	NA	5 (4–24)	6 (4–14)	NA	NA	NA	NA	NA	NA	NA	NA	NA	NA	NA	NA
Gomberawalla *et al.* ^62^	116.78 (79–197)	118.25 (57–218)	NA	NA	1.80 (1–3)	1.75 (1–3)	NA	NA	NA	NA	NA	NA	58.4 (38–102)	58.5 (44–95)	72.3 (24–125)	74.1 (33–108)	NA	NA
Lakdawala *et al.* ^63^	45±20.5	42±18.2	NA	NA	NA	NA	NA	NA	NA	NA	NA	NA	59.7±19.7	58.6±22.3	69.2±22.8	68.3±24	65.4±29.6	69.1±26.3
Muir and Rice^64^	104.6 (68–165)	90.7 (40–139)	32.1 (10–70)	34.3 (10–75)	NA	NA	NA	NA	NA	NA	NA	NA	48.3	59.1	NA	NA	NA	NA
Morales-Conde *et al.* ^66^ ^b^	69.93 (51–88)	61.80 (36–92)	7.66 (0–40)	17.80 (0–150)	NA	NA	34.1±8.9	49.3±12.2	22.1±7.9	35.9±10.2	12.9±4.3	20.1±5.2	56.9±14.9	53.3±13.7	NA	NA	NA	NA
Porta *et al.* ^65^	60±15	61±11	NA	NA	NA	NA	2.1 (0–7)	2.0 (0–6)	1.4 (0–7)	1.8 (0–7)	0.6 (0–4)	0.7 (0–5)	48.9±3.1	48.3±2.9	56.9±2.9	56.5±3.2	59.6±4.6	59.9±4.1
Mauriello *et al.* ^67^	86±16	72±15	45±16	22±12	3±2	5±3	2.8±0.3	3.4±0.6	1.9±0.6	1.8±0.4	0.5±0.4	0.6±0.3	59.2±19.3	59.7±19.9	66.1±24.6	65.1±24.4	67.5±27.2	67.2±26.8
Amiki *et al.* ^68^	148.7±22.6	120.2±25.9	0 (0–1385)	0 (0–367)	3 (3–7)	3 (3–3)	NA	NA	NA	NA	NA	NA	NA	NA	105.9±39.3	109.7±40.0	101.1±51.9	105.3±60.6
Tranchart *et al.* ^69^	65 (55–80)	50 (45–60)	NA	NA	3 (3–3)	2 (2–2)	NA	NA	NA	NA	NA	NA	69.5 (53.2–93.6)	73.3 (60–92.3)	69.8 (54.8–87.3)	73.4 (58.1.9–95.7)	NA	NA
Khidir *et al.* ^70^	74.5±26	68.3±26	NA	NA	3.4±0.9	4±1	NA	NA	NA	NA	NA	NA	NA	NA	67.2 ±18.7	67.7±23.1	NA	NA
Park *et al.* ^71^ ^a^	NA	NA	NA	NA	NA	NA	4.0±1.5	4.3±1.5	3.4±1.0	3.5±1.3	2.8±1.1	2.6±1.1	80.3±25.3	65.9±26.5	90.0±29.8	75.2±29.9	NA	NA

Values are represented as Means±SD or Means (Minimum–Maximum).

a VAS ranged from 0 to 10.

b VAS ranged from 0 to 100.

CLSG conventional laparoscopic sleeve gastrectomy; EWL excess weight loss; NA not available; POD, postoperative day; SLSG single-incision laparoscopic sleeve gastrectomy; VAS, Visual Analog Scale.

**Table 7:** Summary of the complications of studies that were included in the meta-analysis

	Incisional hernia (*n*)		Stricture/obstruction (*n*)		Postoperative bleeding (*n*)		Wound problem (*n*)		Leak (*n*)		Reoperation (*n*)		GERD (*n*)	
References	SLSG	CLSG	SLSG	CLSG	SLSG	CLSG	SLSG	CLSG	SLSG	CLSG	SLSG	CLSG	SLSG	CLSG
Saber *et al.* ^57^	0	0	0	0	0	0	0	0	0	0	0	0	0	0
Park *et al.* ^58^	0	0	0	0	0	0	0	0	0	0	0	0	0	0
Nguyen *et al.* ^59^	0	0	0	0	0	0	1	0	0	0	0	0	1	0
Delgado *et al.* ^60^	0	0	0	0	2	1	0	0	0	0	2	1	0	0
Sucher *et al.* ^61^	0	0	0	0	1	1	0	0	1	0	2	1	0	0
Gomberawalla *et al.* ^62^	0	0	0	0	0	1	0	0	0	0	0	1	0	0
Lakdawala *et al.* ^63^	5	0	0	0	0	0	2	0	2	1	0	0	15	15
Muir and Rice^64^	0	0	0	0	1	0	0	1	0	0	1	0	0	0
Morales-Conde *et al.* ^66^	0	0	0	0	0	0	0	0	0	0	0	0	0	0
Porta *et al.* ^65^	0	0	0	0	0	2	1	1	0	0	0	1	0	0
Mauriello *et al.* ^67^	0	4	0	0	1	2	2	7	0	0	0	0	0	0
Amiki *et al.* ^68^	0	0	0	0	0	0	1	1	1	0	1	0	1	0
Tranchart *et al.* ^69^	5	1	0	0	7	4	0	0	8	8	0	0	0	0
Khidir *et al.* ^70^	0	0	2	4	2	2	2	2	1	1	0	0	0	0
Park *et al.* ^71^	1	0	0	3	0	2	3	2	0	0	4	3	22	40

CLSG, conventional laparoscopic sleeve gastrectomy; GERD, gastroesophageal reflux disease; SLSG, single-incision laparoscopic sleeve gastrectomy.

#### Comparison of intraoperative data

**Figure 3:**
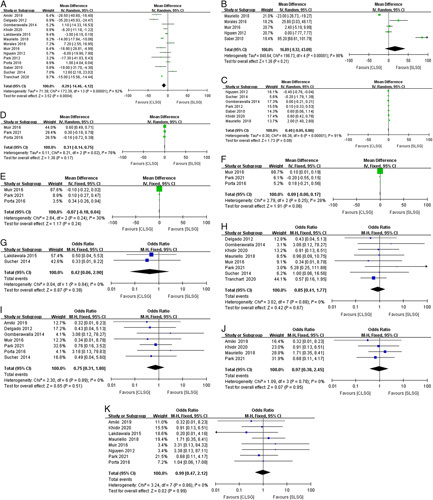
Forest plot. (A) Operative time. (B) Estimated blood loss. (C) Length of stay. (D) Pain Visual Analog Scale score on postoperative day 1. (E) Pain VAS rating on postoperative day 2. (F) Pain Visual Analog Scale score on postoperative day 3. (G) Leak. (H) Postoperative bleeding. (I) Reoperation. (J) Stricture/obstruction. (K) Wound problem.

Intraoperative data included the operative time and estimated blood loss. Fourteen studies reported the operative time in the SLSG and CLSG groups. The operative time was significantly shorter in the CLSG than in the SLSG (*P*=0.0004, Fig. 3A). Heterogeneity was high (*I* ^2^=92%). The operative time showed no change after removing the study by Tranchart and colleagues, resulting in a heterogeneity of 87%. Five studies reported estimated blood loss and no significant differences were observed (*P*=0.21). The heterogeneity was high (*I* ^2^=98%, Fig. 3B), and the results showed no changes after removing the study by Saber and colleagues, generating a heterogeneity of 95%.

#### Comparison of immediate postoperative data

The immediate postoperative data consisted of the length of stay, postoperative analgesia, and postoperative complications. In the comparison of length of stay, there were no statistical differences between SLSG and CLSG (*P*=0.08, Fig. 3C). For postoperative analgesia, three studies reported pain VAS score on POD 1 and 2. The pain VAS score on POD 1 in the CLSG was significantly higher than in the SLSG after removing the study of Porta and colleagues which had the largest heterogeneity among these studies (*P*<0.01, *I* ^2^=29%). The pain VAS score on POD 3 showed a *P*-value of 0.06 between SLSG and CLSG (Figs. 3D–F). This may demonstrate the advantage of SLSG over CLSG in terms of postoperative pain. Regarding postoperative complications, there were no statistical differences in the leak (*P*=0.38), postoperative bleeding (*P*=0.67), reoperation (*P*=0.51), stricture/obstruction (*P*=0.95), or wound problem (*P*=0.99) (Figs. 3G–K).

#### Comparison of late postoperative data

**Figure 4:**
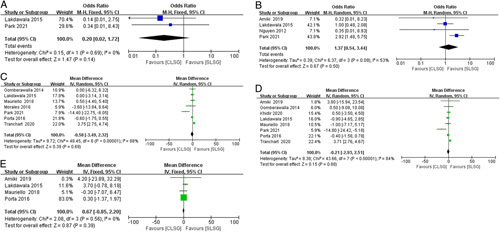
Forest plot. (A) Incisional hernia. (B) Gastroesophageal reflux disease. (C–E) Excess weight loss at 6, 12, and 24 months postoperatively.

Incisional hernia and GERD showed no statistical difference between the SLSG and CLSG (Figs. 4A and B). Moreover, seven studies^60,62–67,69,71^ reported the EWL at 6 months postoperatively, eight studies^62,63,65,67–71^ reported the EWL at 12 months postoperatively, and only four studies^63,65,67,68^ reported the EWL at 24 months postoperatively. The EWL at 6, 12, and 24 months postoperatively showed no statistical differences between the SLSG and CLSG (Figs. 4C–E).

## Discussion

LSG has been well accepted for treating severe morbidly obese patients as it shows the advantages of relative simplicity, good weight loss, and a reduction in hunger sensation by promoting the secretion of glucagon-like peptide-1 and peptide-YY and decreasing ghrelin^72^. The development of laparoscopic surgical techniques has led to a dramatic increase in bariatric surgical procedures, and the number of trocars used in LSG procedures reduced from seven trocars^73^ to four to six trocars^74^. Since 2008, SLSG has only used one or two trocars in total and was initially used for bariatric surgery. Currently, there is no consensus on the safety and surgical outcomes of SLSG and CLSG in the treatment of morbid obesity. Studies on LSG seldom reported the reasons for trocar placement, and these surgeries were reported based on their experiences with no standardization. Therefore, this study was designed to answer the following questions: (1) how many trocars are utilized in the published studies on LSG, (2) how the trocars are placed in these studies, and (3) the safety comparison between LSG involving one to two trocar(s) and multiple trocars.

For the first question, we searched the literatures on trocar number used in the studies on SLSG and CLSG. The majority of LSG (35 studies) were performed based on CLSG involving three or more trocars, and 11 studies were performed based on SLSG involving one (10 studies) or two trocars (one study). In the CLSG studies, the majority were performed using four and five trocars, especially the latter. Among the studies on SLSG, 21 studies utilized periumbilical trocar placement, and four studies utilized trocar placement in the left upper abdomen. Periumbilical trocar placement could yield a better cosmetic appearance as the incision could be covered by the umbilicus. For some morbidly obese patients with an abdominal drop or a high abdominal thickness, trocar placement of the left upper abdominal pattern could provide direct access to the surgical site, avoid digestive interposition, and guarantee an optimal axis for the endoscopic stapler^19^. In this study, we summarized the trocar placement pattern in all the included publications, and the trocar placement in the CLSG procedures was divided into two types, designated IT and Z patterns. Most trocars were arranged in an IT pattern. We proposed the following reasons for this phenomenon: (1) The first trocar was placed at a position above the umbilicus in those with an XU distance less than 25 cm^2^, and then the other trocars were placed above the first trocar to obtain a better surgical field. (2) The observation trocar and the operation trocars were in line with the triangulation, and the IT pattern could avoid the trocar clashing as it generated a distance of 5 cm or more between the two operation trocars. (3) As the target organ was the stomach, the external trocar was more convenient for the surgery than the internal trocar close to the head position.

For the Z pattern, we proposed the following reasons to explain this phenomenon: (1) It was more commonly seen in the subjects with an XU of more than 25 cm, where the trocars should be placed in a position that was comparatively far from the umbilicus and close to the target organ. This facilitated to the visualization of the target organs from the operation and the observation trocars^2^. (2) To avoid trocar clashing, the shortest distance between two trocars should be less than 5 cm. To avoid a close distance between the trocar and the inferior costal margin, and in line with the biomechanical principles, the external trocar should be lower than the internal trocar. (3) The trocar beneath the right costal margin was the liver retractor trocar, and the trocar beneath the left costal margin was used to pull the greater gastric curvature. The trocar at the left side of the umbilicus was used to fold the gastric wall to facilitate the entry of the stapler from the trocar at the right side of the umbilicus. After the gastrectomy, an operation trocar at the right body side was used for suturing and fixation of the cutting margins.

According to the predominant body side of the trocar placement, the trocar placement pattern can be divided into three types: right-predominant, left-predominant, and central type. For the right-predominant type, the trocar beneath the right costal margin was utilized to retract the liver to expose the surgical field. The trocar between the observation trocar and the trocar beneath the right costal margin was used for gastrectomy and subsequent suturing^22,56,75^. The trocar beneath the left costal margin was used to drag the gastric wall to assist the application of the stapler. For the left-predominant type, a left costal trocar was used to retract the liver, and a trocar near the umbilicus on the left side was used to retract the gastric wall. The right trocar was used to place the stapler for final suturing. The central type usually involves five trocars that are suitable for young surgeons as they have fewer operational difficulties. However, this method is not commonly used. Recently, some studies have reported techniques involving three ports with satisfactory safety, which is technically demanding for surgeons^76–78^.

For the third question, single-site laparoscopic surgery, which serves as an evolution derived from classic laparoscopic surgery, is performed by utilizing the same single incision to perform the surgery, which subsequently removes the resected tissues. This technique has great aesthetic benefits, along with decreased postoperative pain and fewer wound-related complications^79,80^. Indeed, there are some disputes regarding the efficiency and safety of SLSG and CLSG. Although two meta-analyses have focused on the comparison between the two methods, these data do not seem to resolve the disputes^81,82^. For the intraoperative indices, unlike the previous studies^81,82^, our data showed that the operative time in the CLSG was significantly shorter than that in the SLSG (*P*<0.05). This was mainly associated with the newly included cohort studies. In addition, few studies have focused on this, and the majority of centers were not experienced in SLSG procedures. Indeed, the learning curve and operation time of SLSG were relatively longer than those of CLSG. With advances in SLSG, its operative time may be similar to that of CLSG in the future. Moreover, there might be bias in the operative time due to various definitions. Therefore, high-quality RCTs with large sample sizes are required to validate this. In contrast, there were no differences in the estimated blood loss between the CLSG and SLSG. For the immediate postoperative indices, there were no statistical differences between the hospital stay and early-stage complications (*P*>0.05). Interestingly, postoperative pain in CLSG was significantly higher than that in the SLSG after removing the studies with high heterogeneity. In addition, there was no statistical difference in the pain VAS score on POD 3 between SLSG and CLSG (*P*=0.06), and less pain may be in the SLSG than that in the CLSG with the increase in the sample size. In the future, more studies with larger sample sizes will be required. The incidence of hernia between the SLSG and CLSG was similar, as the port near the umbilicus in the CLSG was used to remove the dissected gastric tissues, yielding a similar incidence of hernia as that of SLSG^83^. The other postoperative indices were similar, with no statistically significant differences.

There are really some limitations for our study. First, the reasons for trocar placement were not mentioned in most of the included studies. Second, no definite trocar placement pattern was illustrated in these studies due to a lack of cohort studies on trocar placement. Third, subgroup analysis was not performed to the trocar number and placing pattern, as most studies only focused on the technical exploration of the SLSG and CLSG, with rare comparative studies on the efficiency of CLSG using different trocars. Fourth, we only did the systematic review rather than the meta-analysis for the trocar placing pattern in this study, as there are no teams giving definition to the trocar placing pattern. In the future, more studies focusing on trocar placement and a number of trocars are required to further explain this.

## Conclusions

Based on the systematic analysis of trocar placement in SLSG and CLSG, we summarized the trocar placement patterns in these surgeries, which could provide evidence for the appropriate trocar placement for LSG. Meta-analysis showed that, compared with CLSG, SLSG triggered a good cosmetic appearance, with slight attenuation of the postoperative pain. SLSG was associated with a longer operative time. In contrast, the CLSG and SLSG were similar in terms of perioperative complications and postoperative weight loss. In the future, rigorous RCTs with large sample sizes will be required to further illustrate whether SLSG would replace CLSG in bariatric surgery.

## Ethical approval

All analyses were based on previously published studies, thus no ethical approval is required.

## Consent

All analyses were based on previously published studies, thus no patient consent is required.

## Sources of funding

This study was supported by the National Natural Science Foundation of China (grant numbers: 82070869, 81900705).

## Author contribution

Z.J.: data curation, formal analysis, writing – original draft. Z.Z.: investigation, data curation, writing – original draft. T.F.: investigation, writing – review and editing. Y.C.: visualization, writing – review and editing. G.Z.: formal analysis, writing –review and editing. M.Z. and S.H.: conceptualization, writing –review and editing. All authors approved the final version of the manuscript.

## Conflicts of interest disclosure

The authors declare that they have no financial conflict of interest with regard to the content of this report.

## Research registration unique identifying number (UIN)

1. Registry used: PROSPERO.

1. Unique Identifying number or registration ID: CRD42022370245.
2. Hyperlink to your specific registration (must be publicly accessible and will be checked): https://www.crd.york.ac.uk/PROSPERO/display_record.php?RecordID=370245

## Guarantors

Mingwei Zhong and Sanyuan Hu.

## Data availability statement

All original data are available upon reasonable request to the corresponding authors.

## Provenance and peer review

Not commissioned, externally peer-reviewed.
